# Decoding Chronic Charcot Arthropathy: Molecular Mechanisms, Predictive Biomarkers, and Emerging Therapies

**DOI:** 10.2106/JBJS.OA.25.00261

**Published:** 2026-03-18

**Authors:** Osama Embaby, Afdhal Bin Asmadi, Aiman Binte Asmadi, Mohamed Elalfy

**Affiliations:** 1 Department of Trauma and Orthopaedics, Sandwell and West Birmingham Hospitals NHS Trust, Birmingham, UK; 2 Birmingham Heartlands Hospital, Birmingham, UK; 3 Mansoura University Hospitals, Mansoura, Egypt; 4 Department of Orthopedic Surgery, Mansoura University, Mansoura, Egypt

## Abstract

» Chronic Charcot arthropathy results from the convergence of genetic predisposition (OPG/RANKL/RANK polymorphisms), metabolic disturbances (AGEs, vitamin D deficiency affecting 84.2% of patients), and inflammatory dysregulation (RANKL/OPG axis, IL-17 family cytokines), explaining why only 0.08-1% of diabetic neuropathy patients develop this devastating complication.

» The receptor activator of nuclear factor kappa-B ligand (RANKL)/receptor activator of nuclear factor kappa-B (RANK)/osteoprotegerin (OPG) axis dysregulation drives excessive osteoclastogenesis and bone resorption in the acute phase, while impaired Wnt/β-catenin signaling and advanced glycation end products-modified collagen compromise healing quality in the chronic phase, resulting in malunion and permanent deformity.

» Magnetic resonance imaging is the best non-invasive imaging modality for differentiating chronic Charcot from osteomyelitis (single bone involvement beneath ulcer, sinus tract, and abscess favor infection; periarticular distribution favors Charcot), though bone biopsy remains the gold standard when diagnostic uncertainty persists.

» Current management relies primarily on mechanical interventions (accommodative footwear, bracing, surgical reconstruction for unbraceable deformities), but emerging molecular therapies targeting RANKL (denosumab), pro-inflammatory cytokines (IL-17 inhibitors), and Wnt pathway (romosozumab) show promise for disease modification.

» Integrated risk stratification models combining genetic risk scores, serum biomarkers (RANKL/OPG ratio, vitamin D levels), and clinical factors can identify high-risk individuals (AUC 0.89), enabling targeted preventive interventions including vitamin D supplementation, prophylactic off-loading, and potentially pharmacological prevention.

## Introduction

### Definition and Clinical Significance

Diabetes mellitus is a major global health problem, affecting roughly 11% of adults worldwide^1^. Charcot neuro-osteoarthropathy (CNO) is a severe complication of long-standing diabetes, characterized by progressive bone and joint destruction in the foot and ankle that can culminate in deformity and disability^2^.

CNO typically starts with an acute inflammatory phase (erythema, edema, and warmth), followed by a chronic consolidated phase—Chronic Diabetic Charcot Arthropathy (CDCA)—in which fractures heal in malalignment, producing rigid deformity such as the classic rocker-bottom foot^3-6^. These deformities increase plantar pressures, ulcer risk, and subsequent infection/osteomyelitis, driving amputation and excess mortality, with major consequences for quality of life and healthcare costs^7-10^.

### Rationale and Aim of This Review

Although acute Charcot mechanisms have been widely discussed, important determinants of chronic deformity and individual susceptibility remain underintegrated. Recent work highlights (1) genetic variation in the osteoprotegerin (OPG)/receptor activator of nuclear factor kappa-B ligand (RANKL)/receptor activator of nuclear factor kappa-B (RANK) axis; (2) epigenetic signatures (microRNAs and DNA methylation) that favor osteoclastogenesis; (3) diabetes-specific metabolic insults such as advanced glycation end products (AGEs) accumulation and prevalent vitamin D deficiency; and (4) dysregulated, ultimately inadequate Wnt/β-catenin signaling during attempted repair. This review synthesizes these molecular drivers with downstream biomechanical failure and practical diagnostic and therapeutic implications.

### Epidemiology and Burden of Disease

Reported prevalence of CNO in diabetes ranges from 0.08% to 13%, reflecting differences in populations and case ascertainment^11,12^. Incidence has been reported around 0.3% to 0.85% in type 2 diabetes^1^, and absolute case numbers continue to rise with increasing diabetes prevalence^13^. Outcomes remain poor: 5-year mortality exceeds 25% and is higher when ulceration and amputation occur^8,9^. CNO-related deformity markedly increases ulceration risk and contributes substantially to healthcare expenditure, including large cost estimates for diabetic foot complications in England^10,14^.

Recent national data sets underline why chronic Charcot deformity is an increasingly relevant problem for health systems. Rising diabetes prevalence increases the absolute pool at risk, even if individual incidence varies by setting and ascertainment^1,13^. For example, NHS England has reported record numbers of people living with diabetes, and diabetic foot complications account for substantial resource use, including inpatient admissions and high downstream costs for ulceration, infection, and amputation^1,10^. Because established deformity is a major driver of recurrent ulceration and readmission, preventing chronic malalignment (through early recognition, off-loading, and risk stratification) has practical implications beyond the acute inflammatory episode^14^.

### Genetic and Epigenetic Influences on Charcot Arthropathy Development

Despite widespread neuropathy and repetitive trauma, fewer than 1% of neuropathic patients develop CNO, implying additional susceptibility factors^15^. Genetic and epigenetic mechanisms that regulate bone turnover and inflammation are increasingly implicated^16,17^.

Genetic studies have identified single-nucleotide polymorphisms within OPG, RANKL, and RANK genes that are enriched in Charcot cohorts. For example, OPG 245T/G and 1217C/T polymorphisms have been associated with CNO, plausibly shifting the OPG/RANKL/RANK balance toward excessive osteoclast activation and bone resorption^16^.

Mechanistically, these variants are thought to shift the set point of osteoclast regulation. Even modest reductions in functional OPG or increases in RANKL signaling could magnify the resorptive response to repetitive microtrauma in a neuropathic foot, especially when combined with inflammatory cytokine surges during active disease. The current evidence supports a polygenic susceptibility model, where multiple small-effect polymorphisms interact with metabolic stress and mechanical loading to determine who progresses from neuropathy to structural collapse^16^.

Epigenetic regulation may further “tune” the osteoclastogenic response. Circulating micro-RNA profiling identified dozens of differentially expressed miRNAs in Charcot foot, many linked to monocyte-to-osteoclast differentiation (e.g., miR-451a and miR-144-3p)^17^. Whole-methylome analyses of circulating monocytes also demonstrate differential methylation of genes involved in osteoclast formation, supporting an epigenetically primed inflammatory/bone resorptive phenotype^18,19^.

From a translational perspective, these epigenetic signatures are attractive because they are measurable in blood. Panels of differentially expressed miRNAs could potentially function as noninvasive biomarkers of heightened osteoclastogenic potential, while methylation patterns in circulating monocytes may reflect a primed innate immune state that predisposes to disproportionate bone resorption^17-19^. Although these tools are not yet ready for routine care, they offer a plausible route to earlier risk stratification and to monitoring biological response alongside clinical staging and thermometry.

**Fig. 1:**
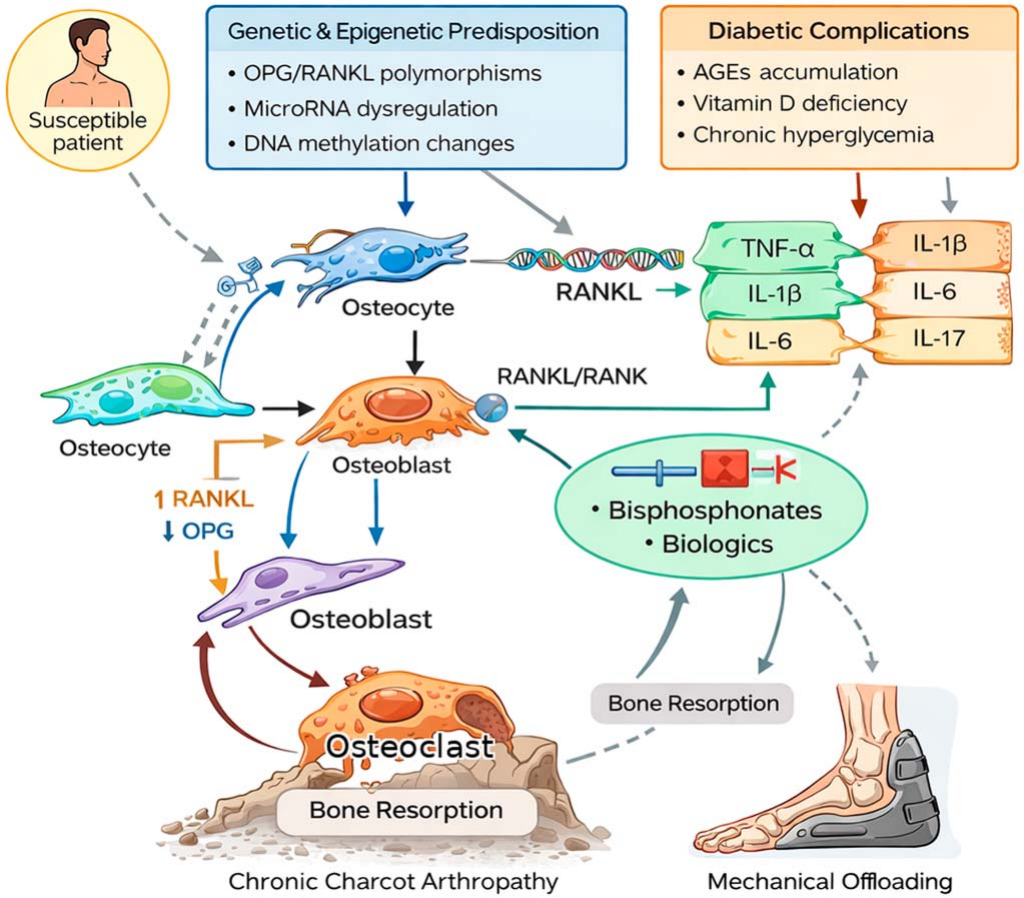
Schematic overview of key molecular interactions implicated in chronic Charcot arthropathy. Genetic/epigenetic susceptibility and diabetes-related metabolic derangements (including AGEs and vitamin D deficiency) promote proinflammatory cytokine signalling (e.g., TNF-α, IL-1β, IL-6, IL-17) and dysregulation of the RANKL/RANK/OPG axis, resulting in increased osteoclastogenesis and bone resorption. This persistent proresorptive milieu, combined with impaired repair responses during consolidation, contributes to structural failure and chronic deformity. AGEs, advanced glycation end products; OPG, Osteoprotegerin; TNF, tumor necrosis factor.

These susceptibility factors likely interact with diabetes-related metabolic stress (hyperglycemia, AGEs, vitamin D deficiency, and pathway dysregulation) to create a “primed” state in which minor mechanical triggers produce disproportionate bone resorption and collapse (Fig. 1).

To help general orthopaedic readers integrate the interacting pathways, Figure 1 presents a schematic summary linking genetic/epigenetic susceptibility and diabetes-related metabolic stress to immune activation and osteoclast-driven bone resorption. In genetically susceptible individuals, hyperglycemia-related insults (including AGEs and vitamin D deficiency) amplify proinflammatory cytokine signaling and shift the RANKL/RANK/OPG balance toward osteoclastogenesis. The net effect is persistent osteoclast activation with impaired, dysregulated repair, which helps explain why minor mechanical triggers can progress to collapse and chronic deformity despite an apparently “low-energy” presentation.

### Transition from Acute to Chronic

The acute phase is dominated by inflammatory osteolysis and joint instability; without early recognition and strict immobilization/off-loading, fragmentation, and subluxation progress rapidly^20-25^. As inflammation settles, bone fragments coalesce and consolidate, but healing occurs within a malaligned architecture, producing rigid deformity, altered load distribution, and long-term ulcer/infection vulnerability^4-7,26^.

The progression from acute to chronic Charcot arthropathy is a critical phase that dictates the long-term structural integrity of the foot^20,21^. The acute stage is dominated by a vigorous inflammatory response, where the foot is erythematous, edematous, and warm to the touch^22^. This inflammatory cascade, driven by proinflammatory cytokines and an imbalanced RANKL/OPG ratio, leads to aggressive bone resorption, osteolysis, and joint destruction^23,24^. If not effectively managed with strict immobilization and off-loading, this process results in fragmentation of subchondral bone, joint subluxation, and dislocation^25^.

As the condition transitions into the chronic phase, the clinical signs of acute inflammation gradually recede^20,21^. The erythema and warmth diminish, and the temperature difference between the affected and contralateral foot normalizes^22^. This quiescent phase is characterized by the body's attempt to repair the damage, leading to the coalescence of bone fragments, sclerosis of bone ends, and the formation of new bone^21^. However, this healing process occurs in the context of a structurally compromised and often malaligned foot^4^. The result is fracture consolidation with significant malunion, leading to permanent, rigid deformities^4,5^. The collapse of the longitudinal arch, resulting in a rocker-bottom deformity, and the abduction of the forefoot are classic hallmarks of the chronic stage^5,6^. These architectural changes redistribute plantar pressures, creating new areas of high mechanical stress that are prone to ulceration and subsequent infection, defining the long-term challenges of managing CDCA^7,26^.

### The Pathophysiology of Chronic Charcot Arthropathy

CDCA reflects an interplay between acute inflammatory bone loss and an impaired, dysregulated repair response in diabetes^2,21,27^. Key drivers include the RANKL/RANK/OPG axis, proinflammatory cytokines (including the Th17/IL-17 pathway), and metabolic factors that degrade bone material properties and fracture healing capacity^23,24,28-32^.

The transition from the acute inflammatory state to the chronic, consolidated phase of Charcot arthropathy is underpinned by a complex interplay of molecular, metabolic, and cellular processes^2,27^. Although the acute phase is defined by rampant inflammation and bone destruction, the chronic phase is characterized by a dysregulated and ultimately inadequate healing response, leading to permanent deformity^21^.

## Molecular Mediators of Inflammation and Bone Resorption

### The RANKL/RANK/OPG System

Osteoclastogenesis is primarily governed by RANKL binding to RANK on osteoclast precursors; OPG acts as a decoy receptor that neutralizes RANKL^23,24^. In active Charcot, RANKL upregulation and a high RANKL/OPG ratio favor aggressive osteoclast activation, driving the osteolysis, fragmentation, and joint disorganization that set the stage for chronic deformity^28,33^.

### Cytokine Dysregulation

Inflamed Charcot tissues show elevated tumor necrosis factor (TNF)-α, IL-1β, and IL-6, which amplify RANKL expression and suppress osteoblast function, creating a self-reinforcing cycle of inflammation and bone loss^27,28,34^. Anti-inflammatory counter signals (e.g., IL-4, IL-10) appear insufficient^34^. More recently, the Th17/IL-17 axis has emerged as an additional osteodestructive pathway: IL-17 family cytokines can intensify local inflammation and synergize with TNF-α and IL-1β to promote osteoclastogenesis and have been reported to rise during active disease and off-loading periods^29^.

Clinically, this cytokine milieu aligns with the observation that active Charcot behaves like an amplified innate immune response rather than a simple mechanical overuse injury. The Th17/IL-17 axis is of particular interest because IL-17 can both augment local inflammation and act synergistically with TNF-alpha and IL-1beta to accelerate osteoclastogenesis, potentially bridging immune activation to structural bone loss^29^. In chronic disease, persistent low-grade inflammatory signaling may continue to influence remodeling during consolidation, even after overt warmth and erythema resolve. This may partly explain why some patients progress to malunion and recurrent breakdown despite apparently adequate off-loading and supports studying inflammatory biomarkers (including IL-17 family cytokines) as adjuncts to clinical staging and thermometry when determining whether the disease process is truly quiescent^27-29,34^.

In the chronic phase, inflammatory cytokines (TNF-alpha, IL-1beta, IL-6, and IL-17) increase RANKL expression and suppress osteoblast activity, tipping remodeling toward osteoclast-driven resorption. In parallel, diabetes-specific metabolic stress (notably AGEs through RAGE signaling and vitamin D deficiency) worsens bone material properties and impairs fracture healing. Although Wnt/beta-catenin signaling may rise as a compensatory anabolic signal during attempted repair, it is often dysregulated and ultimately insufficient, so consolidation occurs in malalignment and rigid deformity.

## Altered Bone Metabolism and Repair in the Chronic Phase

### Advanced Glycation End Products

Chronic hyperglycemia promotes AGE formation and accumulation in bone collagen, producing abnormal cross-linking that increases stiffness and brittleness, reducing the bone’s ability to absorb energy^30^. AGEs also signal through RAGE, increasing oxidative stress, promoting osteoblast dysfunction/apoptosis, and stimulating RANKL expression—thereby worsening both bone quality and the proresorptive cellular milieu^30^.

### Vitamin D Deficiency and Calcium-Phosphate Metabolism

Vitamin D deficiency is common in diabetes and appears particularly prevalent in Charcot cohorts. In one study, 84.2% of Charcot arthropathy patients were vitamin D deficient or insufficient^31^. Deficiency reduces calcium absorption and may contribute to secondary hyperparathyroidism and increased bone resorption, plausibly impairing fracture consolidation and remodeling in CDCA^31,35^.

### The Wnt/β-catenin Signaling Pathway

Wnt/β-catenin signaling promotes osteoblast differentiation and bone formation and is regulated by inhibitors such as sclerostin and Dkk-1^32^. Lower sclerostin/Dkk-1 levels at diagnosis and dynamic changes during off-loading have been interpreted as an endogenous, compensatory anabolic attempt, but one that is ultimately insufficient to prevent deformity^29,32^. More recent work also supports broader Wnt pathway dysregulation in type 2 diabetes and diabetic Charcot bone, suggesting a targetable repair deficit^36^.

These pathway insights also suggest a framework for disease-modifying therapy in the chronic stage. At minimum, they justify a dual approach: (1) suppressing excessive osteoclast activity driven by a high RANKL/OPG ratio and proinflammatory cytokines and (2) supporting an effective anabolic repair response through improved metabolic milieu (glycemic control, vitamin D repletion) and normalization of osteoblast signaling during consolidation^23,24,29,31,32,36^. Importantly, any molecular strategy would need to be paired with strict mechanical off-loading because unchecked load converts biological vulnerability into architectural collapse.

### Biomechanical Consequences and Deformity Patterns

Permanent deformity is the clinical hallmark of CDCA and translates molecular dysregulation into mechanical failure^6^. Midfoot collapse produces a rigid, noncompliant structure; combined with neuropathy and impaired proprioception, this alters gait and concentrates forces on plantar prominences, promoting recurrent microtrauma and skin breakdown^37^.

Functionally, the problem is not only static deformity but also the dynamic load environment. Neuropathy blunts protective pain feedback, so patients continue to walk on an unstable architecture, and abnormal gait concentrates forces at plantar prominences and midfoot joints^37^. This helps explain why midfoot involvement (Sanders/Frykberg patterns) is so strongly linked to rocker-bottom collapse and ulcer risk, while hindfoot/ankle patterns can present with instability and higher reconstructive complexity^38^. In the chronic stage, therefore, the core mechanical goal is to create a stable, plantigrade, braceable foot that spreads pressure across a broad contact area, reducing focal shear and recurrent microtrauma that would otherwise perpetuate skin breakdown and infection risk^4^.

Anatomical patterns are often described using the Sanders and Frykberg classification, in which the midfoot (tarsometatarsal and transverse tarsal joints) is commonly involved and most strongly linked to rocker-bottom deformity, whereas ankle/hindfoot involvement is associated with instability and worse prognosis^38^.

### The Pathway to Ulceration and Infection

**Fig. 2:**
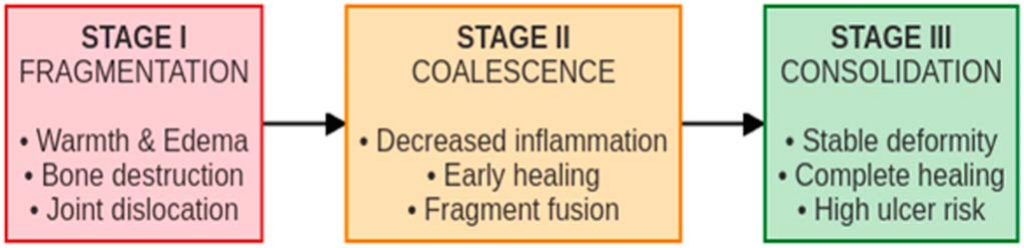
Eichenholtz stages of Charcot arthropathy.

Using the Eichenholtz framework (Fig. 2), Stage III (reconstruction/consolidation) represents a stable but deformed foot in which mechanical factors dominate^4^. High pressure and shear over bony prominences lead to ischemia and ulceration; due to sensory loss, ulcers may be painless and detected late^26^. Ulcers provide a portal for infection that can progress to osteomyelitis, the key driver of subsequent amputation in CDCA^39^.

### Diagnostic Challenges in the Chronic Phase

The main diagnostic dilemma in chronic disease is differentiating quiescent Charcot change from osteomyelitis, particularly when a nonhealing ulcer is present^40,41^. Nuclear medicine scans can be sensitive but nonspecific; bone biopsy remains the reference standard for definitive diagnosis^41^. MRI is often most informative: osteomyelitis typically shows marrow involvement in a single bone beneath an ulcer, whereas Charcot changes are more periarticular and multijoint; MRI also demonstrates high sensitivity and specificity for diabetic foot osteomyelitis in published series^41^.

Clinically, a structured approach can reduce diagnostic delay. Serial skin temperature measurement and comparison with the contralateral foot help confirm quiescence, while a persistent ulcer, systemic features, or rising inflammatory markers increase suspicion for infection. When imaging is equivocal, combining modalities (e.g., MRI for marrow pattern and CT for bony architecture) can help separate periarticular Charcot change from focal osteomyelitis beneath an ulcer and guides the threshold for biopsy when the diagnosis remains uncertain^40-43^. For chronic deformity planning, weight-bearing radiographs and CT can clarify alignment and bony prominences that drive pressure points, which is directly relevant to ulcer prevention and surgical planning^42,43^.

Plain radiographs remain essential for documenting established deformity and progression. Typical chronic findings are summarized as the “6 D’s” (distention, destruction, dislocation, disorganization, debris, density/sclerosis), while CT can better define bony architecture for operative planning when needed^42,43^.

A critical clinical dilemma is differentiating a quiescent chronic Charcot foot from low-grade osteomyelitis, especially with an ulcer. Table I summarizes the key distinguishing features.

**TABLE I:** Differentiating Chronic Charcot Arthropathy from Osteomyelitis

Feature	Chronic Charcot Arthropathy	Osteomyelitis
Clinical	Warm, swollen, but less pain/erythema	Often painful, purulent drainage, sinus tract
MRI	Subchondral cysts, sclerosis, less edema	Diffuse marrow edema, abscess, sinus tract imaging
Radiograph	Bony proliferation, sclerosis, fragmentation	Permeative bone destruction, periosteal reaction
Bone biopsy	Gold standard; shows inflammatory cells, no organisms	Gold standard; shows organisms and inflammation

### Management Strategies for the Chronic Charcot Foot

Because CDCA is usually biologically quiescent but mechanically vulnerable, treatment focuses on lifelong protection of the deformed foot to prevent ulceration, infection, and amputation^44-46^. Custom therapeutic footwear, total contact insoles, and bracing (including Charcot restraint orthotic walker devices for severe deformity) aim to redistribute plantar pressures, accommodate prominences, and reduce focal stress; patient education and daily foot inspection are critical^46^.

A deeper molecular understanding opens the door for disease-modifying therapies. Table II presents potential therapeutic targets to their respective pathways.

**TABLE II:** Potential Molecular Therapeutic Targets in Charcot Arthropathy

Pathway	Target	Potential Drug Class	Example Drug
RANKL/RANK/OPG	RANKL	Monoclonal Antibody	Denosumab
IL-17 pathway	IL-17A	Monoclonal Antibody	Secukinumab
Wnt/β-catenin	Sclerostin	Monoclonal Antibody	Romosozumab
AGE-RAGE	AGEs/RAGE	AGE inhibitors/breakers	Experimental

AGEs, advanced glycation end products; OPG, Osteoprotegerin.

Surgery is reserved for the unbraceable foot, recurrent ulceration despite optimal off-loading, or severe instability. Procedures include exostectomy and deformity correction with arthrodesis/osteotomy to restore a stable, plantigrade, braceable foot but carry substantial risk in this comorbid population^47-50^. Published series report variable outcomes (including reconstruction success rates around the low-to-mid 60% range), with complications such as wound problems, recurrent ulceration, nonunion, and amputation^48,49^. Recent literature suggests increasing the use of reconstruction for hindfoot/ankle Charcot to improve function and reduce downstream complications in selected patients^51^.

In practice, reconstruction aims to restore a plantigrade, braceable foot while minimizing soft-tissue compromise. Contemporary strategies often combine deformity correction with robust fixation (internal, external, or hybrid constructs), prolonged protected weight-bearing, and meticulous ulcer and infection control. Outcomes are heterogeneous and highly selection-dependent: complication profiles commonly include wound breakdown, hardware failure, nonunion, recurrent ulceration, and, in a minority, progression to major amputation^47-50^. These realities reinforce the need for careful patient selection (vascular status, glycemic control, infection exclusion), multidisciplinary team involvement, and realistic shared decision-making when moving beyond lifelong protective bracing^48-51^.

Candidate biomarkers such as circulating miRNA profiles, monocyte methylation signatures, and bone turnover markers (including RANKL/OPG ratio or Wnt pathway inhibitors such as sclerostin/Dkk-1) may help stratify risk, distinguish active from quiescent disease, and identify patients most likely to benefit from targeted antiresorptive or proanabolic strategies alongside mechanical off-loading.

## Conclusion

CDCA is driven by intersecting inflammatory, metabolic, genetic/epigenetic, and biomechanical factors. Acute dysregulation of the RANKL/RANK/OPG axis and cytokine networks (including IL-17) promotes intense osteoclastic resorption; diabetes-related metabolic injury (AGEs, vitamin D deficiency) and impaired anabolic signaling (Wnt/β-catenin) compromise repair and consolidate deformity^28-32,36^. The resulting rigid architecture predisposes to ulceration, infection, and amputation, making lifelong mechanical protection essential and reserving surgery for selected unbraceable cases^26,46,50^. Better integration of molecular biomarkers with clinical staging could enable earlier risk stratification and the development of targeted, disease-modifying therapies beyond mechanical management.

## Funding

This research received no external funding.

## Author Contributions

Conceptualization: Osama Embaby, and Mohamed Elalfy. Methodology: Osama Embaby, and Mohamed Elalfy. Writing—Original Draft Preparation: Osama Embaby, Afdhal Bin Asmadi, and Aiman Binte Asmadi. Writing—Review & Editing: Osama Embaby, Afdhal Bin Asmadi, and Aiman Binte Asmadi. Supervision: Mohamed Elalfy.

## Ethics Approval

Ethical approval was waived for this study as it is a narrative review of existing literature.

## Institutional Review Board Statement

Ethical review and approval were waived for this study, as it did not involve human or animal subjects.
